# Racial and ethnic disparities in surgical amputations following serious musculoskeletal infections in a diverse New Mexico cohort

**Published:** 2019-01-30

**Authors:** Martha L. Carvour, Allyssa Chiu, Kimberly Page

**Affiliations:** ^1^Department of Internal Medicine, Divisions of Epidemiology, Biostatistics, and Preventive Medicine, Albuquerque, NM, United States; ^2^Department of Internal Medicine, Divisions of Infectious Diseases, University of New Mexico, Albuquerque, NM, United States

**Keywords:** amputation, diabetes mellitus, health disparities, musculoskeletal infection

## Abstract

**Background:**

Patients with serious musculoskeletal infections may encounter health disparities across multiple phases of prevention and treatment, including surgical intervention. The purpose of this study was to identify and compare the predictors of surgical intervention and surgical amputation among patients with septic arthritis, osteomyelitis, and infectious myositis in a diverse cohort of patients from New Mexico.

**Methods:**

A retrospective cohort from the University of New Mexico Health System was formed. Patients with septic arthritis, osteomyelitis, and/or infectious myositis who underwent surgical procedures or amputations were compared with those who did not, using predictive multivariable logistic regression modeling. The impact of diabetes mellitus (DM) as a predictor of surgical outcomes was evaluated.

**Results:**

DM was a predictor of both surgical procedures and amputations in a diverse cohort of patients (n = 1694). Diabetes was more common in American Indian/Alaskan Native (AI/AN) patients. However, Black non-Hispanic/African American and Hispanic patients were more likely to undergo amputations, compared to AI/AN patients, even after adjustment for diabetes severity.

**Conclusions:**

Racial and ethnic disparities in infection-related amputation may differ from those observed for diabetes or for general access to surgical management. Interventions intended to prevent or treat serious musculoskeletal infections should consider health disparities that differ across the clinical care process.

## 1. Introduction

### 1.2. Overview

Musculoskeletal infections, including those affecting the joint (septic arthritis), muscle (infectious myositis), and bone (osteomyelitis), are a significant cause of morbidity and disability [1,2]. Treatment of serious musculoskeletal infections often requires surgical drainage or debridement along with delivery of systemic antimicrobial agents.

As the burden of musculoskeletal disease and the associated risks for musculoskeletal infection continues to rise [1,2], characterization of the need for surgical interventions within health systems and populations is needed. At the same time, attention should be directed toward identifying and mitigating potential disparities, if these exist, in treatment access or treatment outcomes across different groups of patients (e.g., across racial/ethnic groups).

### 1.2. Complex clinical processes

Musculoskeletal infections may result from a complex set of factors, including existing pathologies of musculoskeletal structures (e.g., rheumatoid arthritis and traumatic injury) or the presence of comorbid conditions such as diabetes mellitus (DM) or peripheral vascular disease (PVD). The outcomes of musculoskeletal infection may also vary widely, from limited cases requiring single courses of treatment to severe cases requiring multiple courses of treatment or amputation.

We propose that complex clinical processes may result in different yet measurable disparities across the phases of prevention and treatment [3]. For instance, some patients might encounter barriers to preventive care, placing them at high risk for infection, whereas others might experience disparities in treatment outcomes, such as amputation, after infection occurs. Thus, to provide a complete picture of patients’ experiences with the health system, population-based research of musculoskeletal infections might examine and compare key phases in the clinical care process.

To further evaluate this conceptual approach to the study of complex clinical processes, we assessed the records of more than 1600 patients with serious musculoskeletal infections in the University of New Mexico (UNM) Health System. We compared the predictors of surgical intervention, a general proxy of treatment access at the time of infection, and the predictors of surgical amputation, a serious complication of infection. We also evaluated the impact of diabetes control as a measure of preventive care on these results.

We hypothesized that, although surgical procedures and surgical amputations would share some common predictors, the collective set of predictors for each outcome would differ and that such differences would include both clinically plausible patterns (e.g., PVD as a stronger predictor of amputation than surgical procedures overall) and clinically unexplained patterns (e.g., sociodemographic factors such as race/ethnicity, a factor of significant interest in our majority–minority community in New Mexico, as a stronger predictor of amputation). We proposed that clinically unexplained differences may signal important disparities at different points in the care process. For instance, amputation rates by race/ethnicity that are not proportionate to rates for surgical treatment or diabetes control may reflect disparities that are specifically concentrated around the amputation outcome and not fully explained by differential access to care earlier in the process.

## 2. Patients and Methods

### 2.1. Participants

A retrospective cohort of hospitalized adult patients (≥18 years of age) in the UNM Health System was formed. Patients were included in the cohort if they had one or more International Classification of Diseases (ICD, version 9 or 10) code corresponding to osteomyelitis, septic arthritis, and/or infectious myositis between January 1, 2010 and December 31, 2015.

### 2.2. Data collection

For each patient, a series of additional variables was collected. These included sociodemographic characteristics, such as age, sex/gender, and race/ethnicity; data for all procedures which may have been related to the infection, such as biopsy, incision and drainage, amputation, or surgical revision; and other clinical characteristics, including selected comorbidities (e.g., diabetes or renal disease) and medications (e.g., antimicrobial agents, immunosuppressants, or medications used to treat diabetes). Race/ethnicity data extracted from the electronic medical record were based on federal health categories in use at the time the categories were applied [Table 1].

**Table 1:** Characteristics of a diverse university-based cohort of patients (n=1694) with one or more musculoskeletal infection and results from unadjusted predictive models for any surgical intervention and any amputation.

Predictor	n (%) unless otherwise noted	Unadjusted P value and stratum ORs for any procedure (95% CI)^a,b^	Unadjusted P value and stratum ORs for any amputation (95% CI)^a,b^
Age (mean±standard deviation)	53.6±15.2 years	*0.36* 1.00 (0.99, 1.00)	***<0.0001*** **1.02 (1.01, 1.03)**
Sex/gender		***0.02***	***0.0001***
Male	1158 (68.4)	**1.29 (1.04, 1.61)**	**1.76 (1.32, 2.35)**
Female	536 (31.6)	**1.00 (reference category)**	**1.00 (reference category)**
Race/ethnicity (n=1648)		*0.57*	***0.01***
Hispanic	662 (40.2)	0.80 (0.59, 1.09)	**1.50 (1.03, 2.17)**
White non-Hispanic	577 (35.0)	0.77 (0.56, 1.06)	**1.07 (0.72, 1.58)**
American Indian/Alaskan Native	292 (17.7)	1.00 (reference category)	**1.00 (reference category)**
Other (including two or more races)	74 (4.5)	0.85 (0.48, 1.49)	**1.09 (0.54, 2.19)**
Black non-Hispanic/African American	43 (2.6)	0.93 (0.45, 1.89)	**2.72 (1.33, 5.56)**
Primary musculoskeletal infection		***<0.0001***	***<0.0001***
Osteomyelitis	964 (56.9)	**1.00 (reference category)**	**1.00 (reference category)**
Infective/septic arthritis	711 (42.0)	**1.62 (1.31, 2.02)**	**0.23 (0.17, 0.32)**
Infective/septic myositis	19 (1.1)	**0.71 (0.28, 1.79)**	**0.16 (0.02, 1.18)**
Number of musculoskeletal infection types		***<0.0001***	*0.38*
One	1424 (84.1)	**1.00 (reference category)**	1.00 (reference category)
Two or more	270 (15.9)	**3.23 (2.24, 4.67)**	0.86 (0.60, 1.21)
Procedures		N/A	N/A
Any procedure	1185 (70.0)		
Any amputation procedure	308 (18.2)		
Medications			
Immunosuppressants	435 (25.7)	*0.42* 1.11 (0.87, 1.41)	***0.03*** **0.72 (0.53, 0.97)**
Antibiotics	1494 (88.2)	*0.24* 0.82 (0.59, 1.15)	*0.30* 1.24 (0.83, 1.86)
Comorbidities			
Diabetes mellitus	816 (48.2)	***0.01*** **1.33 (1.08, 1.64)**	***<0.0001*** **7.66 (5.55, 10.56)**
Diabetic neuropathy	203 (12.0)	***0.02*** **1.49 (1.06, 2.10)**	***<0.0001*** **5.55 (4.07, 7.58)**
Peripheral vascular disease	119 (7.0)	*0.88* 1.03 (0.69, 1.56)	***<0.0001*** **4.85 (3.30, 7.12)**
Osteoarthritis	295 (17.4)	***0.02*** **1.41 (1.05, 1.88)**	*0.55* 0.90 (0.65, 1.26)
Rheumatoid arthritis	67 (4.0)	*0.76* 1.09 (0.63, 1.87)	***0.03*** **0.35 (0.14, 0.88)**
Renal disease	182 (10.7)	*0.42* 1.15 (0.82, 1.62)	***<0.0001*** **2.90 (2.08, 4.05)**
Cirrhosis	104 (6.1)	*0.87* 0.96 (0.63, 1.48)	***0.12*** **0.63 (0.35, 1.14)**
Obesity	216 (12.8)	*0.64* 1.08 (0.79, 1.48)	*0.61* 1.10 (0.77, 1.58)
Sepsis	462 (27.3)	*0.24* 1.15 (0.91, 1.46)	*0.48* 0.90 (0.68, 1.20)

a Variables with a *P*<0.20 (**bold type**) were eligible for inclusion in the multivariable model.

b Reference categories shown here were selected to permit direct comparison with Tables 2 and 3. OR: Odds ratio, CI: Confidence interval

Data were obtained from the UNM Clinical and Translational Science Center Clinical Data Warehouse (CDW), a service that provides data to research investigators on the UNM campus. All data were de-identified by the CDW before they were issued to the study team. A unique study identification code was assigned to each patient to permit linkage across analytical files. The UNM Institutional Review Board reviewed and exempted this study.

### 2.3. Measures

Musculoskeletal infections were classified into one of the three categories, osteomyelitis, septic arthritis, or infectious myositis, using the corresponding ICD codes. The primary infection for each patient was classified using the first diagnosis listed in chronological order within the study period. The number of musculoskeletal infection types was also recorded (i.e. “one” versus “two or more” from among the three categories).

DM was defined as any ICD code corresponding to any type or any complication of DM, a maximum hemoglobin A1c (HbA1c) of ≥6.5% (48 mmol/mol), and/or a prescription for any DM treatment (e.g., insulin or metformin) during the study period. Diabetic peripheral neuropathy (or other neurological complications of diabetes), PVD, osteoarthritis, rheumatoid arthritis, renal disease (including chronic kidney disease and end-stage renal disease), cirrhosis, obesity, and sepsis were defined as present if any ICD code corresponding to each respective diagnosis was recorded or as absent if no corresponding ICD code was recorded. Similarly, for medications (including DM treatments, immunosuppressants, and antibiotics), patients were classified as having received a medication if any corresponding prescription was recorded and as not having received a medication if no corresponding prescription was recorded.

Two procedure definitions were employed. Patients were classified as having any procedure when one or more facility procedure code (e.g., biopsy, incision and drainage, amputation, or surgical revision) was recorded or as not having a procedure if no corresponding code was recorded. Patients were classified as having an amputation if one or more facility procedure code included the terms “amputation,” “detachment,” or “disarticulation” or as not having an amputation if no such code was recorded.

Procedures and amputations recorded within the first 3 months after the infection diagnosis were included. To account for possible lags between procedures and infection diagnoses (e.g., infection diagnoses based on intraoperative findings or post-operative specimen analysis), procedures or amputations recorded within 10 days before the infection diagnosis were also included in the study.

### 2.4. Analysis

Patients who underwent a procedure were compared with those who did not with respect to a series of sociodemographic and clinical characteristics. This comparison was performed for all procedures and for the subset of procedures which involved an amputation. To achieve this, predictive logistic regression models were developed separately for each of the two surgical outcomes. All variables as shown in Table 1 (except for the procedure variables themselves) were eligible predictors.

Variables with P < 0.20 in an unadjusted model for the surgical outcome were eligible for inclusion in the multivariable procedure. Variables with P < 0.10 in the multivariable model were eligible to remain in the model (P value cutoff higher than 0.05 was used to permit inclusion of more variables of potential clinical interest, which could be evaluated further in future studies with larger datasets). Multivariable models included patients for whom data were available for all variables in the model. C-statistics were computed for each model, as a measure of goodness-of-fit. Models resulting from manual forward and backward selection procedures were compared. For the amputation model, complete information about race/ethnicity was available for 1648 patients (97.3% of the cohort, missing n = 46). The modeling procedure was performed for the set of patients for whom race/ethnicity was defined; we did not impute missing values for race/ethnicity.

Adjusted odds ratios (aORs) and 95% confidence intervals (CIs) for each predictor in the multivariable models were calculated. For dichotomous variables depicting the presence or absence of a diagnosis or treatment, the absence of the diagnosis or treatment served as the reference category. For nominal variables (such as sex/gender) and categorical variables with more than two categories, the category with the lowest odds served as the reference group, unless that category included only a small number of participants (e.g., the infective/septic myositis stratum (n = 19, 1.1% of cohort) of the infection type variable, where osteomyelitis was used as the reference category).

Adjustment for DM severity with HbA1c was used as a proxy for preventive care. However, HbA1c values were not available for all patients in the cohort. Furthermore, this variable was expected to have complex relationships with the other variables in the model. For instance, HbA1c is a potential confounder for some variables (e.g., infection type) and a potential mediator for some others (e.g., DM). For these reasons, HbA1c values were not included in the primary modeling procedures, but *post hoc* adjustments were performed to evaluate the relationships between variables and to generate further hypotheses.

Statistical analyses were performed in SAS version 9.4 (SAS Institute, Cary, NC). Based on existing guidance about statistical power in multivariable regression models, an estimated minimum of 100 procedures was expected to provide adequate power for a multivariable model with up to 10 parameters [4]. Preliminary estimates of amputation frequencies at our hospital suggested that >5 years of data would be needed to attain 200–300 amputations and, therefore, to permit consideration of up to 20–30 parameters. 6 years of data were included here. For the amputation outcome in this study, where the total number of outcomes was approximately 300, up to 30 parameters could be considered.

## 3. Results

### 3.1. Cohort characteristics

**Figure 1:**
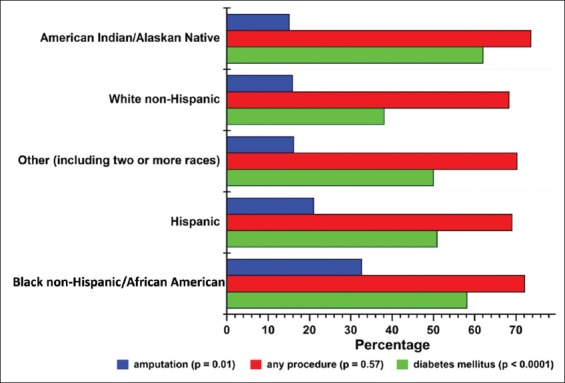
Frequency of diabetes mellitus (DM), any surgical procedure (including amputation), and any surgical amputation by race/ethnicity in a diverse group of patients with serious musculoskeletal infections (n = 1648). P values are from unadjusted logistic regression models for DM, any procedure, or any amputation, where race/ethnicity is the predictor variable in each model.

**Figure 2:**
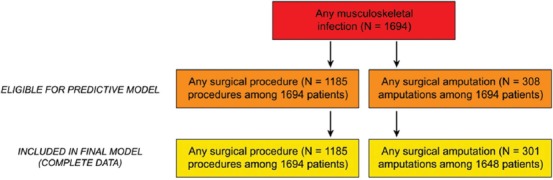
Cohort members who underwent any surgical procedure (including amputation) or any surgical amputation. Any patient with a documented procedure or amputation was eligible for the corresponding predictive model.

The sociodemographic and clinical characteristics of the cohort are summarized in Table 1 and Figure 1. The cohort was diverse with respect to sex/gender, race/ethnicity, and infection type. Women represented almost one-third of the group (n = 536, 31.6%). The most frequently reported race/ethnicity category was Hispanic (40.2%), followed by White non-Hispanic (35.0%), American Indian/Alaskan Native (AI/AN, 17.7%), other (including two or more races, 4.5%), and Black non-Hispanic/African American (2.6%). Figure 2 outlines the number of patients with each surgical outcome who were eligible for the predictive models.

Most patients (n = 1424; 84.1%) had only one of the three infection types recorded, although a significant subset of the cohort (n = 270; 15.9%) had diagnoses corresponding to more than one infection type. Osteomyelitis (n = 964; 56.9%) and septic arthritis (n = 711; 42.0%) were the most common. A majority of patients (n = 1185; 70.0%) underwent a procedure, while 308 patients (18.2%) underwent an amputation.

Almost half (48.2%) of the patients in the cohort met at least one criterion for DM. Within this group, the maximum HbA1c values ranged from 4.0% (20 mmol/mol) to 16.2% (154 mmol/mol), with a mean ± standard deviation of 8.6% (70 mmol/mol) ± 2.7% (6 mmol/mol), from n = 753 patients with DM and at least one HbA1c recorded during the study period.

Figure 1 shows the proportion of patients in each race/ethnicity category who had diabetes or who underwent any procedure or amputation. Overall, AI/AN (62.0%) and Black non-Hispanic/African American (58.1%) patients were most likely to have DM, while Black non-Hispanic/African American (32.6%) and Hispanic (21.0%) patients were most likely to undergo an amputation.

### 3.2. Predictive model for any surgical procedure

Variables with P values and ORs in bold print in Table 1 (second column from right) were eligible for inclusion in the multivariable model predicting the occurrence of a procedure. Variables listed in Table 2 were retained in the final multivariable model. Manual forward and backward selection procedures produced the same model for this outcome.

**Table 2:** Multivariable predictive model for any procedure among patients with one or more musculoskeletal infections (n=1185 procedures among 1694 patients, c-statistic 0.64).

Predictor	Adjusted odds ratio (95% CI)	P value
Sex/gender		*0.04*
Male	1.27 (1.01, 1.58)	
Female	1.00 (reference category)	
Infection type		*<0.0001*
Infective/septic arthritis	1.56 (1.24, 1.96)	
Infective/septic myositis	0.39 (0.15, 1.03)	
Osteomyelitis	1.00 (reference category)	
Number of musculoskeletal infection types		*<0.0001*
Two or more	3.17 (2.15, 4.68)	
One	1.00 (reference category)	
Diabetes mellitus	1.35 (1.07, 1.71)	*0.01*
Diabetes-associated peripheral neuropathy	1.46 (1.00, 2.13)	*0.05*^a^

a Variables with a *P*< 0.10 were eligible for retention in the model. CI: Confidence interval

Male sex, DM, and diabetic neuropathy were all significant independent predictors of a procedure (Table 2). Septic arthritis was associated with the highest independent odds of a procedure compared to the other infection types (aOR = 1.56, 95% CI: 1.24, 1.96; relative to the osteomyelitis reference category). The presence of two or more musculoskeletal infection types was also associated with higher odds of undergoing a procedure (aOR = 3.17, 94% CI: 2.15, 4.68) compared to one infection type.

Adjustment of the model as shown in Table 2 for HbA1c (model with n = 913) attenuated the ORs for DM (aOR 1.16, 95% CI: 0.77, 1.75), DM-associated peripheral neuropathy (aOR = 1.37, 95% CI: 0.93, 2.03), male sex (aOR = 1.19, 95% CI: 0.87, 1.64), and multiple infection types (aOR = 1.77, 95% CI: 1.05, 2.99). In contrast, the OR point estimate for septic arthritis (aOR = 1.81, 95% CI: 1.26, 2.60) increased after this adjustment, while the estimate for septic myositis (aOR = 0.40, 95% CI: 0.10, 1.63) was similar. In this model, a single-unit rise in HbA1c (e.g., 7.0–8.0% or 53–64 mmol/mol) was associated with an aOR of 1.16 (95% CI: 1.08, 1.24) for undergoing a procedure.

### 3.3. Predictive model for any amputation procedure

Variables with P values and ORs in bold print in Table 1 (far right column) were eligible for inclusion in the multivariable model predicting the occurrence of an amputation. Variables listed in Table 3 were retained in the final multivariable model. Manual forward and backward selection procedures produced the same model for this outcome. Use of the age variable with or without natural log transformation also did not affect the final model.

**Table 3:** Multivariable predictive model for any amputation among patients with one or more musculoskeletal infection (n=301 surgical amputations, detachments, or disarticulations among 1648 patients, c-statistic 0.81).

Predictor	Adjusted odds ratio (95% CI)	P value
Sex/gender		*0.002*
Male	1.65 (1.20, 2.29)	
Female	1.00 (reference category)	
Race/ethnicity		*0.04*
Black non-Hispanic/African American	2.94 (1.29, 6.71)	
Hispanic	1.65 (1.10, 2.48)	
Other (including two or more races)	1.10 (0.52, 2.34)	
White non-Hispanic	1.35 (0.87, 2.09)	
American Indian/Alaskan Native	1.00 (reference category)	
Infection type		*<0.0001*
Infective/septic arthritis	0.34 (0.24, 0.47)	
Infective/septic myositis	0.22 (0.03, 1.79)	
Osteomyelitis	1.00 (reference category)	
Diabetes mellitus	4.70 (3.29, 6.72)	*<0.0001*
Diabetic neuropathy	2.17 (1.52, 3.10)	*<0.0001*
Peripheral vascular disease	2.08 (1.35, 3.20)	*0.001*
Rheumatoid arthritis	0.42 (0.15, 1.15)	*0.09*^a^

a Variables with a *P*< 0.10 were eligible for retention in the model. CI: Confidence interval

Osteomyelitis was associated with higher independent odds of amputation compared to the other infection types. DM, diabetic peripheral neuropathy, and PVD were all significant independent predictors of amputation, whereas patients with rheumatoid arthritis were generally less likely to undergo an amputation (Table 3).

Two sociodemographic variables were also significant predictors of amputation, even after adjustment for the other variables as shown in Table 3. Men were more likely to undergo an amputation compared to women (aOR = 1.65, 95% CI: 1.20, 2.29). Meanwhile, Black non-Hispanic/African American (aOR = 2.94, 95% CI: 1.29, 6.71) and Hispanic (aOR = 1.65, 95% CI: 1.10, 2.48) patients were more likely to undergo an amputation compared to AI/AN patients, who had the lowest odds of this outcome in the cohort.

Adjustment of the model shown in Table 3 for HbA1c (model with smaller n = 895) attenuated the ORs for DM (aOR = 1.91, 95% CI: 1.00, 3.62), peripheral neuropathy (aOR = 1.95, 95% CI: 1.34, 2.85), PVD (aOR = 1.89, 95% CI: 1.19, 3.00), and rheumatoid arthritis (aOR = 0.58, 95% CI: 0.20, 1.70). The OR for septic myositis (aOR = 0.16, 95% CI: 0.02, 1.48) decreased further after adjustment, while the OR for septic arthritis (aOR = 0.32, 95% CI: 0.21, 0.47) was similar. A single-unit rise in HbA1c was associated with an aOR of 1.20 (95% CI: 1.13, 1.28) for undergoing an amputation.

Adjustment for HbA1c did not change the patterns observed with the sociodemographic characteristics in the amputation model. Male sex was still associated with more than a 60% increase in the odds of an amputation (aOR = 1.69, 95% CI: 1.17, 2.46). Black non-Hispanic/African American (aOR = 3.83, 95% CI: 1.50, 9.76), Hispanic (aOR = 1.64, 95% CI: 1.05, 2.55), other race/ethnicity (aOR = 1.14, 95% CI: 0.48, 2.71), and White non-Hispanic (aOR = 1.15, 95% CI: 0.70, 1.89) patients still had increased odds of amputation compared to AI/AN patients after adjustment for HbA1c.

## 4. Discussion

The mainstays of treatment for serious musculoskeletal infections include administration of systemic antimicrobial therapy and, when appropriate, surgical drainage or debridement of the infected anatomic structures. In this cohort of patients with osteomyelitis, septic arthritis, and/or infectious myositis, surgical procedures were common (70.0%) and amputation occurred in nearly one-fifth of cases (18.2%).

Several clinical characteristics of this cohort were notable. DM was a prevalent condition (48.2%). Both DM and DM-associated peripheral neuropathy significantly increased the odds of undergoing either a surgical procedure or an amputation. These findings underscore the risk of musculoskeletal infections in patients with DM (2,5) and may signify a diversity of mechanisms, by which DM increases this risk (e.g., neuropathic and non-neuropathic processes). Furthermore, after adjustment for HbA1c, the impact of both DM and peripheral neuropathy was attenuated in both models, suggesting that the impact of these diagnoses on procedural occurrence may be mediated by (or closely correlated with) the degree of hyperglycemia.

As expected, the type of infection also had a significant impact on the odds of undergoing a procedure or amputation. Septic arthritis was a stronger predictor of procedures than osteomyelitis or infectious myositis, whereas osteomyelitis was a stronger predictor of amputation than septic arthritis or infectious myositis. These patterns likely reflect clinical practice standards, in which septic arthritis often poses an urgent indication for surgical drainage [5], whereas severe or persistent osteomyelitis (e.g., in the bones of the feet) may require surgical resection or amputation [2].

Men had higher odds of both surgical procedures and amputations compared to women. It is unknown whether men in the cohort may have had higher rates of other unmeasured predictors, such as musculoskeletal trauma. Interestingly, adjustment for DM severity using HbA1c largely ameliorated the increased odds of a procedure among male patients. However, this was not observed in the amputation model, in which men had persistently higher odds, regardless of DM severity.

Race/ethnicity was a significant predictor for amputations but not for surgical procedures overall. This is consistent with evidence elsewhere that DM and its complications, including amputation, exert disproportionate impacts across different racial and ethnic groups [6-9]. Our study appears to confirm the presence of significant racial/ethnic disparities in surgical amputation among patients with serious musculoskeletal infections. Importantly, however, adjustments for DM severity using HbA1c did not eliminate this apparent disparity, suggesting that race/ethnicity may not be related entirely to preventive DM care.

The disparate odds of amputation by race/ethnicity also cannot be extrapolated to the odds for developing a musculoskeletal infection in the first place. In this cohort of patients with serious musculoskeletal infections, 17.7% of patients identified as AI/AN - a proportion exceeding the state population percentage of 8.8% [10] - even though AI/AN patients had the lowest apparent odds of undergoing an amputation. AI/AN populations have the highest prevalence of DM in the United States, in New Mexico, and in this study (Figure 1) [6,11]. This disproportionate impact of DM on AI/AN populations may lead to an increased risk for musculoskeletal infections - a disparity unto itself - even when the amputation odds are not elevated.

Importantly, our study cannot account for other competing risks for amputation, including other complications of DM (such as all-cause mortality or cardiovascular events that could preclude surgical management), which could impact the relative odds of amputation across racial and ethnic groups in our study. In New Mexico, diabetes-related mortality rates are higher among AI/AN populations than other racial and ethnic groups [11].

Taken together, these findings reinforce the view that disparities may differ in important ways across the clinical phases of care. For instance, AI/AN patients may have higher risks for infection (e.g., due to higher rates of diabetes), lower rates of amputation, and higher rates of other competing risks, such as mortality. Meanwhile, Black non-Hispanic/African American and Hispanic patients may encounter pronounced disparities in amputation rates, regardless of access to surgical services (e.g., no racial/ethnic disparities observed for surgical interventions overall) or the degree of diabetes management (e.g., no reduction in amputation disparities observed after adjustment for DM or HbA1c).

Future work should be directed toward understanding apparent racial and ethnic disparities across the full spectrum of care for patients who are at risk for DM, musculoskeletal infection, and amputation. These studies should account for the severity of diabetes and its complications, including the competing risks for certain endpoints, such as amputation.

Our study has several strengths. The cohort consisted of over 1600 patients - over 1100 of whom underwent at least one procedure and over 300 of whom underwent an amputation - over 6 years of data collection. The cohort was also diverse with respect to race and ethnicity. Less than half (35.0%) of patients identified as White non-Hispanic, whereas 40.2% identified as Hispanic and 17.7% identified as AI/AN.

This study also has several limitations. All data were collected from a single university-based health system, which serves as a referral center throughout the state of New Mexico. Clinical data from other referring sites, including those affiliated with Indian Health Services facilities within the state, were not available for this study. We acknowledge that some prior diagnosis or procedure codes, if recorded at a referring center and not also recorded at our facility, could have been misclassified as absent in our analysis. Based on our clinical experience with these infections, we anticipate that this was less likely to occur for diagnoses that require active management in hospitalized patients (e.g., DM) and for surgical procedures themselves among patients who were admitted or referred to our center for management. In any case, future work in conjunction with other referring sites is needed to confirm the relationships between providers and assess the relationship of these data across sources.

The study is also limited by the retrospective and de-identified nature of data collection. Specific details about the nature of each infection, including detailed information about infection sites; the timing or duration of DM; specific procedural, radiological, or microbiological findings; or other clinical impressions which may have contributed to surgical management were not available. Similarly, information about individual or cultural perceptions about specific surgical procedures, including amputation, was not available.

Furthermore, our study relied on ICD-based coding and facilities procedure coding, which may have been incomplete for some patients or which may have underestimated bedside or clinic-based procedures. However, a comparison of ICD-based coding for DM and laboratory-based or medication-based definitions of DM in our study showed that the rate of underdiagnosis of DM by ICD-based coding alone was minimal (of 1694 patients, only n = 56 or 3.3% of the cohort met laboratory-based or medication-based criteria for DM without a corresponding ICD code and were added to the DM group using this expanded definition).

Finally, in our study, only procedures which occurred within the first 3 months of the infection were included. For this analysis, we intended to study these early or initial outcomes (e.g., when procedures may have been most likely to occur at a single center and when competing or intervening events or diagnoses that may impact surgical indications were minimized). However, it is important to note that the resulting models may not extend to more chronic or relapsing infections.

## 5. Conclusions

In summary, our findings suggest that patients in different racial and ethnic groups may encounter different health disparities at different clinical stages in the prevention and treatment of serious musculoskeletal infections. Although AI/AN patients in this cohort were most likely to have DM, Black non-Hispanic/African American and Hispanic patients were most likely to undergo an amputation. Ongoing research is needed to understand the processes leading to these apparent disparities.
